# Progress in the clinical application of immune checkpoint inhibitors in small cell lung cancer

**DOI:** 10.3389/fimmu.2023.1126582

**Published:** 2023-03-29

**Authors:** Jiahui He, Qinyong Hu

**Affiliations:** Department of Oncology, Renmin Hospital of Wuhan University, Wuhan, Hubei, China

**Keywords:** small cell lung cancer, immune checkpoint inhibitors, combined immunotherapy, clinical trials, CTLA- 4, PD1，PD-L1

## Abstract

Small cell lung cancer (SCLC) is a refractory cancer with poor prognosis due to its aggressive malignancy and high rates of metastasis, recurrence and drug resistance. These characteristics have also greatly impeded the identification of new treatment methods and drugs. The traditional model of SCLC treatment that has been reliant on platinum combined with etoposide for decades has been superseded by the emergence of immune checkpoint inhibitors (ICIs), which have shown significant therapeutic effects and broad application prospects as a monotherapy. This has led to the evaluation of ICIs with different mechanisms of action and their use in combination with radiotherapy or a variety of molecular targeted drugs to achieve synergy, complementary advantages, and reduce adverse reactions. Here, we review the progress in the use of ICIs as a monotherapy or in combination therapy for SCLC and consider the current limitations of these approaches as well as prospects for future developments.

## 1 Introduction

Small cell lung cancer (SCLC), which accounts for approximately 15% of all types of lung cancer, is a neuroendocrine tumor with rapid growth, early metastasis and poor prognosis (1). Platinum and etoposide (EP)-based systemic chemotherapy has long been considered the first-line treatment for extensive-stage SCLC. Chemotherapy is effective in early-stage SCLC, but the vast majority of patients will rapidly relapse and die within a few months (2).

Immunotherapy has become an important strategy for the treatment of tumors. Cancer immunotherapies include tumor vaccines, cytokines, chimeric antigen receptor T cell immunotherapy (CAR-T), and immune checkpoint inhibitors (ICIs), which have become a focus of research in recent years (3). ICIs eliminate tumors by inhibiting the immune escape of tumor cells and enhancing the immune response of T cells (4). ICIs have been positively correlated with tumor mutation burden (5). SCLC is a smoking-related disease characterized by a high tumor mutation burden, indicating that SCLC may be highly sensitive to ICI-based immunotherapy (2). The ICIs used to treat SCLC include inhibitors of programmed cell death 1 (PD-1), programmed cell death ligand (PD-L1) and cytotoxic T lymphocyte-associated antigen 4 (CTLA-4) (6). Numerous clinical studies are ongoing to further explore the role of ICIs as adjuvant or neoadjuvant therapy for lung cancer patients.

The field of SCLC research was widely considered to be a “forbidden zone” until the emergence of ICIs offered the potential for more efficient and less toxic modes of immunotherapy both alone and in combination with radiotherapy or a variety of molecular targeted drugs. Here, we review the clinical trials of ICIs as monotherapy and in combination therapy for SCLC (**Table 1**), and discuss the progress in this field as well as the limitations and prospects for future developments that will pave the way for improved outcomes for patients with SCLC.

**Table 1:** Completed and ongoing clinical trials of immunotherapy for SCLC.

Study ID	Trial identifier	Phase	Patients	Treatment methods	Estimated primary completion date	Status	Reference
CA184-041	NCT00527735	II	Untreated SCLC	Ipilimumab+paclitaxel/carboplatin vs. paclitaxel/carboplatin	December 2011	Completed	(7)
CA184-156	NCT01450761	III	ES-SCLC	Ipilimumab+etoposide/platinum vs. etoposide/platinum	May 17, 2017	Completed	(8)
KEYNOTE-028	NCT02054806	IB	PD-L1-ES-SCLC	Pembrolizumab	April 30, 2021	Completed	(9)
KEYNOTE-158	NCT02628067	II	Advanced SCLC	Pembrolizumab	June 18, 2026	Ongoing	(10)
KEYNOTE-604	NCT03066778	III	ES-SCLC	Pembrolizumab+EP vs. placebo+EP	September 21, 2021	Completed	(11)
REACTION	NCT02580994	II	Untreated ES-SCLC	Etoposide and cis/carboplatin ± pembrolizumab	December 2023	Ongoing	(12)
–	NCT02402920	I	SCLC	Pembrolizumab and concurrent chemoradiotherapy or radiation therapy	July 31, 2023	Ongoing	(13)
KEYLYNK-013	NCT04624204	III	ES-SCLC	Pembrolizumab+concurrent chemoradiation therapy followed by pembrolizumab ± olaparib vs. concurrent chemoradiation therapy	October 28, 2027	Ongoing	(14)
CheckMate 032	NCT01928394	I/II	Recurrent SCLC	Nivolumab vs. nivolumab+ipilimumab	April 30, 2023	Ongoing	(15)
CheckMate331	NCT02481830	III	Relapsed SCLC	Nivolumab vs. chemotherapy	August 22, 2022	Completed	(16)
CheckMate 451	NCT02538666	III	ES-SCLC	Nivolumab vs. nivolumab+ipilimumab vs. placebo	November 11, 2021	Completed	(17)
IMpower133	NCT02763579	I/III	Untreated ES-SCLC	Carboplatin+etoposide ± atezolizumab	July 8, 2022	Completed	(18, 19)
SKYSCRAPER-02	NCT04256421	III	Untreated ES-SCLC	Atezolizumab+carboplatin+etoposide ± tiragolumab	March 21, 2024	Ongoing	(20)
CASPIAN	NCT03043872	III	Untreated ES-SCLC	Durvalumab ± tremelimumab in combination with Platinum-based chemotherapy	December 30, 2022	Completed	(21)
BALTIC	NCT02937818	II	Platinum Refractory ES-SCLC	Durvalumab+tremelimumab followed by durvalumab monotherapy	December 29, 2023	Ongoing	(22)
–	NCT02701400	–	Relapsed SCLC	Tremelimumab+durvalumab combination ± radiation	August 7, 2020	Completed	(23)
PAVE	NCT03568097	II	Advanced SCLC	Avelumab combined with chemotherapy	April 2023	Ongoing	(24)
QUILT-3.055	NCT03228667	IIb	Previously received treatment with PD-1/PD-L1 ICIs	Avelumab	December 2023	Ongoing	(25)
JAVELIN Medley	NCT02554812	Ib/II	SCLC	Avelumab+utomilumab	February 28, 2023	Ongoing	(26)
–	NCT05429866	II	SCLC	Immune checkpoint inhibitor(s) (ICI) alone or in combination with chemotherapy or targeted therapy	December 1, 2024	Ongoing	(27)
CAPSTONE-1	NCT03711305	III	Untreated ES-SCLC	Carboplatin+etoposide with or without Adebrelimab	December 2023	Ongoing	(28)
–	NCT03041311	II	Untreated ES-SCLC	Carboplatin, Etoposide, and Atezolizumab With or Without Trilaciclib	October 29, 2020	Completed	(29)
–	NCT02514447	Ib/IIa	ES-SCLC Receiving Topotecan Chemotherapy Previously	Trilaciclib and topotecan or placebo and topotecan	October 4, 2021	Completed	(30)
–	NCT02499770	Ib/IIa	Untreated ES-SCLC	Trilaciclib/placebo + carboplatin/etoposide	February 22, 2019	Completed	(31)

## 2 CTLA-4 inhibitors

CTLA-4 is a negative regulator of T cell activation. As the first ICIs for SCLC (32), CTLA-4 inhibitors include ipilimumab and tremelimumab. Ipilimumab is a human monoclonal IgG1 antibody against CTLA-4, which blocks the immunosuppressive interaction between CTLA-4 and its ligands on cells (CD80/CD86) to promote the activation and proliferation of T cells, and enhance anti-tumor immune function (33). Tremelimumab is a fully human monoclonal IgG2 antibody that is still in preclinical testing.

### 2.1 Ipilimumab combined with chemotherapy

The CA184-041 study (7) was a randomized control trial of 130 treatment-naive patients with extensive-stage small-cell lung cancer (ES-SCLC) who were randomly allocated to a staged treatment group (paclitaxel and carboplatin combined with ipilimumab), a concurrent chemotherapy group (paclitaxel and carboplatin combined with ipilimumab) and a control group (paclitaxel and carboplatin). While the objective response rate (ORR) and immune-related progression-free survival (irPFS) were increased in the phase-therapy ipilimumab group, there were no significant improvements in the concurrent chemotherapy group. Compared with the control group, the staged ipilimumab regimen improved irPFS (HR = 0.64; *P* = 0.03), but not in the concurrent ipilimumab regimen (HR = 0.75; *P* = 0.11). Median irPFS was 5.3 months for control, 6.4 months for staged ipilimumab, and 5.7 months for concurrent ipilimumab regimens. However, treatment-related grade III/IV immune adverse events (AEs) were more common in the ipilimumab arm. Interpretation of the results of this study is limited by its small sample size and the availability of only preclinical data on ipilimumab plus chemotherapy.

The efficacy and safety of ipilimumab or placebo combined with platinum and etoposide in the treatment of newly diagnosed ES-SCLC patients have been evaluated in a phase III clinical study (CA184-156) (8). Among 1,132 patients randomly assigned to receive ipilimumab or placebo, the median OS was 11.0 months and 10.9 months (HR = 0.94; 95% CI: 0.81–1.09; *P* = 0.3775), respectively, and PFS was 4.6 months and 4.4 months (HR = 0.85; 95%CI: 0.75–0.97, *P* = 0.016), respectively. These results showed that there was no significant improvement in the primary endpoint of OS compared with chemotherapy alone, so the difference in the secondary endpoint PFS could not be considered statistically significant. Diarrhea, rash, and colitis were more common with chemotherapy plus ipilimumab, while other treatment-related AEs were of similar frequency and severity in the two groups. Treatment-related discontinuation was higher with ipilimumab (18% vs. 2% with placebo). Five treatment-related deaths occurred in the ipilimumab group and two in the placebo group. The toxicity of ipilimumab combined with carboplatin and etoposide (ICE) in the treatment of ES-SCLC was also found in the study reported by Edurne et al. (34). It is not clear why ipilimumab was not more effective than etoposide+ platinum-based chemotherapy, although one possible explanation is that ipilimumab does not effectively stimulate peripheral T cell activation, and thus activated T cells that could effectively enhance antitumor immune responses are not present in the tumor microenvironment.

## 3 PD-1 and PD-L1 inhibitors

PD-1 and PD-L1 play an important role in regulating T cell function to maintain protective immunity and immune balance, homeostasis and tolerance. The combination of PD-1 and PD-L1 has an immunosuppressive effect, transmits negative signals, inhibits T cell proliferation, cytokine production and cytolytic function, and maintains the balance of the immune system (35). Currently, pembrolizumab and nivolumab are PD-1 inhibitors that are widely studied in the field of SCLC, and PD-L1 inhibitors include atezolizumab, durvalumab and avelumab (36).

### 3.1 Pembrolizumab

Pembrolizumab is a highly selective humanized monoclonal antibody that binds to the PD-1 receptor and directly blocks the interaction between PD-1 and its ligand, thereby enhancing the function of tumor-directed T cells and mediating tumor destruction (37).

#### 3.1.1 Pembrolizumab in monotherapy

The KEYNOTE-028 study (9) included 24 patients with SCLC who failed to respond to standard chemotherapy and had PD-L1 expression confirmed by immunohistochemistry. The results of this study showed that the ORR was 33%, the median OS was 7.7 months, the PFS was 1.9 months, and the 1-year survival rate was 37.7%. This study confirmed that pembrolizumab monotherapy showed promising anti-tumor activity and was well-tolerated in the treatment of PD-L1-positive, previously treated SCLC. However, all patients experienced treatment-related AEs, the most common of which were fatigue (n = 7) and cough (n = 6). The incidence of immune-related toxicities was 12.5% (3/24), including immune thyroiditis, infusion site reactions, cytokine release syndrome, and colitis. Toxicity was consistent with that observed previously for pembrolizumab therapy in other solid tumors. Subsequently, the KEYNOTE-158 study (10) was conducted to better identify biomarkers that would more accurately identify SCLC patients who might respond to pembrolizumab. In patients with relapsed or metastatic SCLC who received pembrolizumab monotherapy (regardless of PD-L1 expression), the ORR was 18.7%, median OS was 8.7 months, and median PFS was 2.0 months. In both studies, pembrolizumab had a favorable safety profile, which was consistent with the safety profile of this monotherapy in other tumor types. Chung et al. (38) conducted a pooled analysis of these two studies, and the median OS and PFS (7.7 and 2.0 months, respectively) were similar to those observed in the subgroup populations of the two studies. In this pooled analysis, pembrolizumab showed promising antitumor activity and durable clinical benefit, supporting the use of pembrolizumab monotherapy in third-line or later treatment for patients with SCLC. Pembrolizumab was recently approved by the U.S. Food and Drug Administration for patients with previously treated metastatic SCLC who had disease progression during, or after platinum-based chemotherapy on the basis of the KEYNOTE-028 and KEYNOTE-158 studies (39).

#### 3.1.2 Pembrolizumab combined with chemotherapy

Studies have shown that ICIs combined with chemotherapy drugs can activate immune cells. ICIs can maintain the activation state of T cells after stimulating specific anti-tumor immune cells with high frequency and low dose chemotherapy. Therefore, ICIs combined with chemotherapy can produce a synergistic effect and enhance the anti-tumor immune response; this raises the possibility of eliminating drug-resistant tumor cells, which is not possible with any of the current treatment modalities (40). The randomized, double-blind, phase III KEYNOTE-604 study (11) compared pembrolizumab/placebo plus etoposide and platinum (EP) in previously untreated patients with ES-SCLC. A total of 453 participants were randomized to receive pembrolizumab plus EP or placebo plus EP. The estimated 12-month PFS was 13.6% with pembrolizumab plus EP and 3.1% with placebo plus EP. The incidence of AEs from any cause was 76.7% and 74.9% for grade 3-4 and 6.3% and 5.4% for grade 5 in the pembrolizumab + EP and placebo + EP groups, respectively. The results showed that adding pembrolizumab to standard first-line EP significantly improved PFS in patients with ES-SCLC (HR = 0.75; 95%CI = 0.61–0.91; *P* = 0.0023), and no unexpected toxicities were observed. Many ongoing studies, such as the REACTION study (NCT02580994), are also evaluating pembrolizumab in combination with standard chemotherapy regimens for the first-line treatment of SCLC (12). Overall, these data support the benefit of pembrolizumab in SCLC, adding to a growing body of evidence supporting the value of immune checkpoint inhibitors (ICIs) in this historically difficult-to-treat cancer.

#### 3.1.3 Pembrolizumab combined with radiation therapy

In preclinical models, ionizing radiation induces PD-L1 expression in tumor and stromal cells, along with an increase in myeloid-derived suppressor cells (41, 42). In addition, tumor-associated antigens released after radiation-induced cell death may be highly immunogenic, thereby enhancing the anti-tumor efficacy of systemic immunotherapy agents, even at distant tumor sites (43–45). Anti-PD-L1 inhibitors combined with radiotherapy have shown synergistic effects in xenograft models of pancreatic, colon, and breast cancer (43–45). Therefore, the combination of the two can enhance the local and systemic anti-tumor immune response and improve the success rate of treatment (46).

A phase I trial (NCT02402920) (13) evaluated the safety of pembrolizumab combined with thoracic radiation therapy (TRT) after induction chemotherapy in patients with ES-SCLS. The results showed that pembrolizumab combined with TRT was well-tolerated, and the incidence of serious AEs was low. However, studies with a longer follow-up time and larger sample size are still needed to improve outcomes compared with immunotherapy or TRT alone. However, in European subclinical trials (47), the OS of this combination treatment group and the TRT alone treatment group were 8.4 months and 8 months, respectively, and the PFS were 6.1 months and 4 months, respectively, showing the advantage of the combination therapy. The phase I trial to evaluate the safety and efficacy of this combination regimen laid a solid foundation for future prospective studies.

Ongoing studies include comparisons of pembrolizumab plus concurrent chemoradiotherapy followed by pembrolizumab with or without olaparib are ongoing in patients with newly diagnosed LS-SCLC (KEYLYNK-013, NCT04624204) (14) in addition to phase II studies of pembrolizumab and lenvatinib plus chemotherapy in the treatment of ES-SCLC. We expect these studies to provide more evidence that will guide the use of pembrolizumab in the treatment of SCLC.

### 3.2 Nivolumab

Nivolumab is the first fully human IgG4 antibody approved by the FDA and the first to be studied clinically in non-small cell lung cancer. In 2018, nivolumab was approved for second-line treatment of SCLC, marking a great leap forward in the treatment of SCLC and indicating that immunotherapy is gradually changing the overall treatment layout of this disease (48).

In 2020, the CheckMate 032 study (15), which provided the latest body of data, demonstrated that nivolumab monotherapy and nivolumab+ ipilimumab showed anti-tumor activity with durable efficacy and manageable safety in previously treated SCLC patients. Ready et al. (49) further reported the efficacy of nivolumab monotherapy as a third-line or late-stage treatment for relapsed SCLC. The ORR was 11.9% (95% CI: 6.5–19.5), and the 12-month and 18-month overall survival rates were 28.3% and 20.0%, respectively, with an incidence of grade 3–4 AEs of 11.9%. These results demonstrate that nivolumab has durable efficacy and is well-tolerated as third-line or late-stage treatment for relapsed SCLC. However, in the CheckMate 331 study (16), 569 patients with SCLC who relapsed after first-line chemotherapy were randomized to receive nivolumab or chemotherapy (topotecan or amrubicin). The results showed that nivolumab was not effective in improving the survival of patients with relapsed SCLC compared with chemotherapy (median OS: 7.5 months vs. 8.4 months; HR = 0.86; 95% CI = 0.72–1.04; *P* = 0.11), and no new safety signals were observed. In addition, the CheckMate 451 study (17) compared the efficacy of nivolumab monotherapy versus nivolumab combined with ipilimumab in patients with ES-SCLC. The results showed that for patients who did not progress on first-line chemotherapy, the combination group (HR = 0.92; 95% CI: 0.75–1.12) and single-agent group (HR = 0.84; 95% CI: 0.69–1.02) did not significantly improve OS compared with placebo and with the same safety profile. Although the trial results indicated that maintenance nivolumab monotherapy or combination therapy was not effective for patients with ES-SCLC, patients in the combination group showed a trend toward benefit. At present, several relevant clinical trials are still ongoing and expected to yield more data on optimizing the combination regimen.

Tumor cells have multiple immune signaling pathways, and inhibition of only one may lead to compensatory upregulation of other immune checkpoint molecules. This mechanism has a significant limiting effect on ICI monotherapy; however, two different types of ICIs can be combined to regulate T cells by acting on different sites. Thus, the synergistic anti-tumor effects of the ICI combination can stimulate the production of a large number of specific T cells in the early stage, and restore the immune function of exhausted T cells in the late stage (50). In this regard, the combination of nivolumab, a PD-1/PD-L1 inhibitor, and ipilimumab, a CTLA4 inhibitor, shows encouraging promise (15).

### 3.3 Atezolizumab

Atezolizumab was the first PD-L1 inhibitor to be studied in SCLC. Studies have shown that atezolizumab combined with conventional chemotherapy regimens can significantly prolong OS and PFS as the first-line treatment of ES-SCLC, with comparable safety. Liu et al. (18) reported the latest OS data based on a large sample population. The IMpower133 study (18, 19) evaluated the efficacy and safety of atezolizumab combined with carboplatin plus etoposide as first-line treatment for ES-SCLC. A total of 403 patients with ES-SCLC were randomized to atezolizumab+carboplatin+etoposide or placebo+carboplatin+etoposide. The results showed that at a median follow-up of 13.9 months, the median OS was 12.3 months in the atezolizumab group and 10.3 months in the placebo group (HR = 0.70, 95%CI: 0.54–0.91; *P* = 0.007), and the median PFS was 5.2 months and 4.3 months, respectively (HR = 0.77; 95%CI: 0.62–0.96; *P* = 0.02) (1). The IMpower133 study was terminated early because the efficacy was so good that OS and PFS had already reached positive results at the time of the interim analysis. Mansfield et al. (51) evaluated AEs in the IMpower133 study and found that grade 3–4 AEs were similar in the two groups. The IMpower133 trial is the first clinical study to achieve dual positive endpoints in the first-line treatment of ES-SCLC in more than 30 years. As a result, this new regimen has been adopted as the first-line treatment for ES-SCLC, representing an important milestone in SCLC immunotherapy. Indeed, based on this study, atezolizumab combined with carboplatin/etoposide chemotherapy is now recommended as a class I regimen for the first-line treatment of ES-SCLC in the 2019 edition of the NCCN SCLC clinical guidelines. Atezolizumab has also become the first immunotherapy agent approved for the first-line treatment of SCLC.

Tiragolumab is a human monoclonal antibody that targets T cell immunoreceptor with immunoglobulin and immunoreceptor tyrosine-based inhibitory motif domains (TIGIT), which is expressed by natural killer (NK) cells in the majority of tumors and competes with the costimulatory molecule CD226 (DNAM-1) for binding to the ligands CD155 and CD112. A number of preclinical trials have shown that anti-TIGIT antibody and anti-PD-1/PD-L1 antibody function synergistically to provide anti-tumor effects and enhance the anti-tumor responses. Consequently, this combination has become an immune checkpoint of great interest after CTLA-4 and PD-1/PD-L1. The phase III trial SKYSCRAPER-02 (20) compared tiragolumab+ atezolizumab+ carboplatin+ etoposide (CE) with placebo+atezolizumab+CE in chemotherapy-naive patients with ES-SCLC. Tiragolumab was found to provide no additional benefit when added to atezolizumab and chemotherapy. The PFS and OS observed in the control group supported the results of the IMpower133 trial and further confirmed the validity of this combination as the standard of care in the first-line treatment of patients with ES-SCLC. The SKYSCRAPER-02 study will continue with OS analysis and biomarker analysis.

### 3.4 Durvalumab

Durvalumab, a selective human IgG1 monoclonal antibody directed against PD-L1, exerts antitumor activity by preventing immune escape mediated by the PD-L1 pathway (52).

#### 3.4.1 Durvalumab combined with chemotherapy

In the CASPIAN study (21), durvalumab, a selective human IgG1 monoclonal antibody directed against PD-L1, was shown to exert anti-tumor activity by preventing immune escape mediated by the PD-L1 pathway. Similar to the IMpower133 trial, the EP regimen plus durvalumab significantly improved median OS compared with the EP regimen alone (13 months vs. 10.3 months, HR = 0.73, 95% CI: 0.59–0.91, *P* = 0.0047). The ORR was also improved (79.5% vs. 70.3%), although the incidence of grade 3–4 AEs and AE mortality were similar in the two groups. Based on the results of this study, the 2020 National Comprehensive Cancer Network Clinical Practice Guidelines (3^rd^ edition) recommended durvalumab plus EP as the preferred first-line treatment for patients with ES-SCLC. The U.S. FDA subsequently approved this regimen for first-line treatment of ES-SCLC in March 2020.

#### 3.4.2 Durvalumab combined with tremelimumab

Inhibitors of CTLA-4 and PD-1/PD-L1can restore anti-tumor immune responses, resulting in long-term benefits in a substantial proportion of patients treated. ICI combination therapy is an emerging treatment option (53). The meta-analysis by Francesco et al. suggested that the current PD-1/CTLA-4 inhibitor combination therapy has a limited effect in advanced NSCLC patients with high and/or low PD-L1, but may be an effective and tolerable option in the PD-L1-negative subgroup (54). In ES-SCLC, studies have shown that PD-1/PD-L1 inhibitors combined with chemotherapy are safer and more effective than chemotherapy alone, whereas PD-1/PD-L1 inhibitors combined with CTLA-4 inhibitors did not improve the efficacy (55).

The combination regimen of durvalumab+ tremelimumab (D+T), which acts *via* the same mechanism, has attracted widespread attention. The phase II clinical trial NCT02937818 (22) initially confirmed the good safety and reliable anti-tumor activity of this “golden partner” group. A preliminary analysis of the phase III CASPIAN study (56), in which durvalumab+ tremelimumab+ platin-etoposide was compared with platin-etoposide alone, were reported in 2021, and the analysis of the total OS of durvalumab+ platin-etoposide and platin-etoposide alone were updated after 11 months of follow-up. Patients were randomized to receive durvalumab+ tremelimumab+ platinum-etoposide (268 patients), durvalumab+ platinum-etoposide (268 patients), or platinum-etoposide (269 patients). Durvalumab+ tremelimumab+ platinum-etoposide did not significantly improve overall OS compared with platinum-etoposide treatment (HR = 0.82,95% CI: 0.68–1.00; *P* = 0.045), with a median total OS of 10.4 months (95% CI: 9.6–12.0) vs. 10.5 months (9.3–11.2). Compared with the platinum-etoposide group, the durvalumab+ platinum-etoposide group had a significant improvement in OS (HR = 0.75, 95% CI: 0.62–0.91; *P* = 0.0032), with a median OS of 12.9 months (95% CI: 11.3–14.7) vs. 10.5 months (9.3–11.2). Durvalumab+platinum-etoposide showed a sustained improvement in OS, but the addition of tremelimumab to durvalumab did not significantly improve prognosis. These results support durvalumab+platinum-etoposide as first-line treatment for ES-SCLC. This study and the IMpower133 study provide compelling evidence that PD-L1 monoclonal antibody combined with chemotherapy is a successful first-line treatment strategy for ES-SCLC.

#### 3.4.3 Durvalumab combined with radiation therapy

A phase II study (NCT02701400) (23) of patients with relapsed SCLC who had received ≤2 lines of prior therapy were randomly assigned to two groups: (1) Group A: received durvalumab (D) tremelimumab (T), but did not receive stereotactic body radiation therapy (SBRT); (2) Group B: immune susceptibility SBRT (9 Gy × 3 F) was performed on a selected tumor site, before patients received D/T. The median PFS times of groups A and B were 2.1 months and 3.3 months (HR = 2.44, 95% CI: 0.75–7.93, *P* = 0.122), respectively, and the median OS times were 2.8 months and 5.7 months (HR = 1.50, 95% Cl: 0.45–4.99, *P* = 0.507), respectively. These studies showed that radiotherapy combined with immunotherapy improved efficacy, although there were no significant difference in the OS and PFS times between the two groups, which is worthy of further exploration in relapsed SCLC. Future studies should take full advantage of the synergy between radiation and immunotherapy in the early stages of disease, while also seeking enrichment strategies for patients who may benefit from immunotherapy.

### 3.5 Avelumab

A phase II study evaluating the safety and efficacy of avelumab+cisplatin or carboplatin+etoposide (NCT03568097) in 55 subjects, with the primary endpoint of 1-year PFS rate, is expected to be completed in April 2023 (24). Another phase IIb multicenter immune-combination study (NCT03228667) is ongoing to validate avelumab in SCLC patients previously treated with PD-1/PD-L1 ICIs (25). Two additional studies (NCT02554812 and NCT05429866) are also ongoing and may lead to the development of new ways to treat patients with SCLC (26, 27).

### 3.6 Adebrelimab

Adebrelimab is a novel humanized IgG4 monoclonal antibody directed against PD-L1. In the phase III CAPSTONE-1 study (28), 462 treatment-naive patients with ES-SCLC were randomized to receive adebrelimab+ chemotherapy (n = 230) or placebo+ chemotherapy (n = 232). The results presented at the American Association for Cancer Research annual meeting in April 2022 showed that adebrelimab significantly improved OS compared with chemotherapy, with a median OS of 15.3 and 12.8 months (HR = 0.72, *P* = 0.0017), respectively, and PFS of 5.8 months and 5.6 months (HR = 0.67, 95% CI 0.54–0.83), respectively. Grade 3 or higher treatment-related AEs occurred in 85.7% and 84.9% of the patients in the two groups, respectively. Hematologic toxicity most common (≥5%) AE in the two groups. At present, chemotherapy is still the main treatment for SCLC. The wide application of immunotherapy and the emergence of various adjuvant therapies is expected lead to new treatment methods that will overcome the problem of chemoresistance.

## 4 Summary and prospect

With the development of molecular biology, several candidate therapeutic targets for SCLC have been reported including poly ADP-ribose polymerase (PARP), enhancer of zeste homologue 2 (EZH2), and delta-like ligand 3 (DLL3). ICIs combined with PARP inhibitors and DLL3-targeted antibody conjugated drugs will become a new direction for the treatment of drug-resistant SCLC (57).

Trilaciclib is a selective, reversible cyclin-dependent kinase 4 and 6 (CDK4/6) inhibitor that reduces bone marrow hematopoietic stem cell depletion during treatment and protects the immune system (58). Based on the results of three phase II clinical trials (NCT03041311, NCT02514447, NCT02499770) (29–31), the U.S. FDA approved Trilaciclib in February 2021 before treatment with platinum-based/etoposide or topotecan-based regimens in adult patients with ES-SCLC to reduce the incidence of chemotherapy-induced myelosuppression (59).

The advent of ICIs has facilitated major breakthroughs in the first- and third-line treatment of SCLC, which is gradually changing the overall therapeutic landscape. ICI monotherapy and combination therapy are now the standard treatment options for patients with SCLC. Extensive research on the immune mechanism and tumor microenvironment has led to a gradual standardization of combined immunotherapy, As an emerging research hotspot, it is hoped that future studies will lead to diversification of strategies using ICIs in combinations that will improve their therapeutic effects in SCLC patients.

## Author contributions

All authors contributed to the article and approved the submitted version.
